# Fast Transient Thermal Analysis of Non-Fourier Heat Conduction Using Tikhonov Well-Conditioned Asymptotic Waveform Evaluation

**DOI:** 10.1155/2014/671619

**Published:** 2014-06-11

**Authors:** Sohel Rana, Jeevan Kanesan, Ahmed Wasif Reza, Harikrishnan Ramiah

**Affiliations:** Department of Electrical Engineering, Faculty of Engineering, University of Malaya, 50603 Kuala Lumpur, Malaysia

## Abstract

Non-Fourier heat conduction model with dual phase lag wave-diffusion model was analyzed by using well-conditioned asymptotic wave evaluation (WCAWE) and finite element method (FEM). The non-Fourier heat conduction has been investigated where the maximum likelihood (ML) and Tikhonov regularization technique were used successfully to predict the accurate and stable temperature responses without the loss of initial nonlinear/high frequency response. To reduce the increased computational time by Tikhonov WCAWE using ML (TWCAWE-ML), another well-conditioned scheme, called mass effect (ME) T-WCAWE, is introduced. TWCAWE with ME (TWCAWE-ME) showed more stable and accurate temperature spectrum in comparison to asymptotic wave evaluation (AWE) and also partial Pade AWE without sacrificing the computational time. However, the TWCAWE-ML remains as the most stable and hence accurate model to analyze the fast transient thermal analysis of non-Fourier heat conduction model.

## 1. Introduction

The classical Fourier heat conduction law follows a property, which is not physical, that is, an infinite thermal wave propagation speed [1–3]. Fourier's law is quite accurate for most common engineering problems. In some cases, the Fourier law cannot explain, such as near the absolute zero temperature, thermal gradient, which is extreme [3]. Non-Fourier conduction accounts for two-phase lag due to thermal wave propagation speed and another one due to relaxation time of electron. D. Y. Tzou proposed a non-Fourier model based on two-phase lag as follows [1, 3, 4]:

(1) $$\begin{array}{c} Q\left(r,t+\chi_{q}\right)=-K_{c}\nabla \theta \left(r,t+\chi_{T}\right), \end{array}$$

where *χ*_*T*_ stands for finite relaxation time for electron phonon and *χ*_*q*_ represents finite thermal wave speed [5].

Conventional solver based on iterative, as fourth-order Runge-Kutta technique, is computationally expensive, though its result is accurate. In contrast, asymptotic waveform evaluation (AWE) technique is much faster solver [6]. Taylor's series was used to expand the AWE transfer function [7–10]. Then the required moments are found from the coefficient of Taylor's series [9–11]. AWE technique is able to solve up to three-dimensional models [12]. However, the limitation of AWE model is that this model cannot predict the temperature responses accurately as it is ill-conditioned moment matching [6, 13]. Then Loh et al. [6] developed technique, which is based on the AWE to remove the instability of AWE, called partial Pade AWE. In partial Pade AWE, selected poles and residues from any heat imposed boundary are used to calculate the temperature responses of the whole system. However, this technique is unable to predict the actual temperature. This prompted the present study to use another technique, which is based on well-conditioned asymptotic waveform evaluation (WCAWE).

WCAWE algorithm was introduced by Slone et al. [13, 14] to solve frequency domain electromagnetic problem based on the FEM. In this model, a correction term was introduced during the poles and residues calculation. Maximum likelihood (ML) was used successfully to calculate poles and residue to bring down the instability of AWE and also partial Pade AWE. Later, Liu et al. solved Fourier heat conduction by using the WCAWE technique [15]. The model proposed in the present work is based on the WCAWE algorithm to analyze the non-Fourier thermal problem. To avoid the singularity problem, our proposed model is embedded with Tikhonov regularization technique [16] to further enhance stable responses. Hence, the present model is recognized as TWCAWE. Later, we developed another well-conditioned scheme, called TWCAWE using the mass effect (TWCAWE-ME) that reduces the computational time compared to TWCAWE using the ML scheme (TWCAWE-ML).

## 2. Non-Fourier Model

The non-Fourier model with two-phase lag is shown in (2) in the case of fast thermal conduction. This model is converted into a normalized hyperbolic equation given by

(2) ∂2φ∂ς2+∂2φ∂ψ2+ZT∂3φ∂τ∂ς2+ZT∂3φ∂τ∂ψ2  =∂φ∂τ+Zq∂2φ∂τ2,

where

(3) $$\begin{array}{c} \varphi =\frac{\theta -\theta_{0}}{\theta_{w}-\theta_{0}},\;\;\tau =\frac{t}{l^{2}/\hbar },\;\;\varsigma =\frac{x}{l},\\ \psi =\frac{y}{h},\;\;Z_{T}=\frac{\chi_{T}}{l^{2}/\hbar },\;\;Z_{q}=\frac{\chi_{q}}{l^{2}/\hbar }, \end{array}$$

where *ħ* is the thermal diffusivity.

FEM meshing is performed to generate the rectangular element. The nodes of the element are denoted by *j*, *k*, *m*, and *o*. Applying FEM based on the Galerkin weighted residual technique of (2), we obtain

(4) ∫Δ[N]T[∂2φ∂ς2+∂2φ∂ψ2+ZT∂3φ∂τ∂ς2+ZT∂3φ∂τ∂ψ2    −∂φ∂τ−Zq∂2φ∂τ2]∂ς∂ψ=0.

The above non-Fourier heat conduction equation shown in (4) is converted into a linear equation based on the Galerkin weighted residual technique, as shown in

(5) $$\begin{array}{c} D\overset{..}{\theta }+V\overset{.}{\theta }+K\theta =F\left(t\right), \end{array}$$

where mass matrix *D* ∈ *R* ^*N*×*N*^, damping matrix *V* ∈ *R* ^*N*×*N*^, stiffness matrix *K* ∈ *R* ^*N*×*N*^, and *F*(*t*) ∈ *R* ^*N*^. The elemental matrices obtained from (4) are as follows:

(6) D=AZq36[4212242112422124],K=ς6∗ψ[21−1−212−2−1−1−221−2−112]+ψ6∗ς[21−1−212−2−1−1−221−2−112],V=A36[4212242112422124]+ZTς6∗ψ[21−1−212−2−1−1−221−2−112]+ZTψ6∗ς[21−1−212−2−1−1−221−2−112].

The Laplace transform of (5) is shown below:

(7) D(s2θ(s)−sθ′(0)−θ′(0)) +V(sθ(s)−θ(0))+Kθ(s)=F,s2Dθ(s)+s(Vθ(s)−Dθ′(0)) +Kθ(s)−Dθ(0)−Vθ′(0)=F.

The system solution *T*(*s*) can be approximated by using polynomial equation in s-domain, as given by the following equation:

(8) $$\begin{array}{c} \theta \left(s\right)=\overset{\infty }{\underset{n=0}{\sum }}M_{n}s^{n}. \end{array}$$

The moments, *M*_*n*_, are the coefficients of Taylor series expansion about *s* = 0 (Maclaurin series).

## 3. TWCAWE Algorithm

The TWCAWE algorithm is initiated by applying the Laplace transform on (5) where the moments obtained are used to estimate the zero state response (ZSR) and zero input response (ZIR). The moment computation for ZSR and ZSR is shown in (10) and (12), respectively.

### 3.1. Zero State Response

In ZSR, the initial condition is assumed to be zero, *θ*(0) = 0 and *θ*′(0) = 0:

(9) D(s2θ(s)−sθ′(0)−θ′(0)) +V(sθ(s)−θ(0))+Kθ(s)=F,(Ds2+Vs+K)θ(s)=F,(Ds2+Vs+K)(M0+M1s+M2s2+⋯+Mnsn)=F.

By equating the same powers of *s*, the moments are generated from the equation below:

(10) $$\begin{array}{c} KM_{0}=F\\ KM_{1}=-VM_{0}\\ KM_{n}=-\left(DM_{n-2}+VM_{n-1}\right),\;\text{for}\;\;n=2,3,\ldots ,\left(2q-1\right), \end{array}$$

where *q* is the order of Pade approximation.

### 3.2. Zero Input Response

In ZIR, the input force is assumed to be zero, *F* = 0:

(11) D(s2θ(s)−sθ′(0)−θ′(0)) +V(sθ(s)−θ(0))+Kθ(s)=0,(s2D+s(Vθ(s)−Dθ′(0))+K)θ(s) =Dθ′(0)+Vθ(0)(s2D+s(Vθ(s)−Dθ′(0))+K)   ×(M0+M1s+M2s2+⋯+Mnsn)=Dθ′(0)+Vθ(0).

By equating the same powers of *s*, the moments are generated from the equation below:

(12) $$\begin{array}{c} KM_{0}=V\theta \left(0\right)+D\overset{.}{\theta }\left(0\right),\\ KM_{1}=D\theta \left(0\right)-VM_{0},\\ KM_{n}=-\left(DM_{n-2}+VM_{n-1}\right),\;\text{for}\;n=2,3,\ldots ,\left(2q-1\right). \end{array}$$

The moment matching process in AWE is inherently ill conditioned. To overcome this instability, WCAWE was proposed [13]. In our proposed model, to reduce the instability, we removed the singularity problem by changing the stiffness matrix. Consider a well-conditioned approximation problem *Kx* ≈ *y*; the residual ||*Kx*−*y*||_2_ becomes smaller for the proper choice of *x* = (*K*∗*K*)^−1^ *K*∗*y* [16]. The singularity condition of *K*∗*K* is removed by adding *h*_*c*_^2^ *I* term where, after modification, it becomes

(13) $$\begin{array}{c} x={\left(K*K+h_{c}^{2}I\right)}^{-1}*y, \end{array}$$

where *h*_*c*_ is the regulation parameter, which depends on the order of the equation, whereas *I* is the identical matrix. The approximate inverse family is defined by *C*_*h*_ = (*K*∗*K* + *h*_*c*_^2^ *I*)^−1^ *K*. As given in (10) and (12), moment calculation involves inverse of stiffness matrix *K*. The resulting moments, especially higher order moments, are product of lower order moments and, hence, the singularity problem can accumulate the compounded error. Therefore, in TWCAWE, computing moments by the inverse of *K* matrix is now replaced by inverse of *C*_*h*_. Subsequently, the ill-conditioned moments are converted to well-conditioned moments. The new moments, called TWCAWE moments, are obtained from ICAWE moments. The ICAWE moment subspace is *M*_*n*_ = (*u*_1_*, *u*_2_*,…, *u*_n_*) and the TWCAWE moments subspace is denoted as follows: *M*_n_* = (*u*_1_, *u*_2_,…, *u*_*n*_), where the relation between ICAWE and TWCAWE moments is *u*_*n*_ = *u*_n_*/*η*_*n*_ where *η*_*n*_ = ||*u*_n_*||. Modified Gram-Schmidt technique is required to orthonormalize *M*_n_* that gives a more accurate solution [11]. In TWCAWE algorithm, *Z* can be computed as shown in (16):

(14) ProjMa∗Mn∗=(Mn∗·Ma∗)Ma∗,for  a=1,2,…,(n−1),

(15) $$\begin{array}{c} u_{n}=M_{n}^{*}-{\text{Proj}}_{M_{a}^{*}}M_{n}^{*}, \end{array}$$

(16) $$\begin{array}{c} M_{n}=Z^{-1}M_{n}^{*}. \end{array}$$

The nodal moment [*m*] can be extracted from the global moment matrix [*M*] for any arbitrary node *i* as follows:

(17) $$\begin{array}{c} {\left[m_{n}\right]}_{i}={\left[M_{n}^{*}\right]}_{i}. \end{array}$$

The transient response for any arbitrary node *i* can be approximated by using Pade approximation and then further simplified to partial fractions [17], as shown in

(18) θi(s)=m0+m1s+m2s2+⋯+mnsn=d0+d1s+⋯+ds−1ss−11+c1s+⋯+csss=R1s−P1+R2s−P2+⋯+Rss−Ps.

Poles and residues can be found by solving

(19) $$\begin{array}{c} \left[\begin{array}{ccccc} m_{0} & m_{1} & m_{2} & \cdots & m_{q-1}\\ m_{1} & m_{2} & m_{3} & \cdots & m_{q}\\ \cdot & \cdot & \cdot & \cdot & \cdot \\ \cdot & \cdot & \cdot & \cdot & \cdot \\ m_{q-1} & \cdots & \cdots & \cdots & m_{2q-2} \end{array}\right]\left[\begin{array}{c} c_{s}\\ c_{s-1}\\ \cdot \\ \cdot \\ c_{1} \end{array}\right]=-\left[\begin{array}{c} m_{s}\\ m_{s-1}\\ \cdot \\ \cdot \\ m_{1} \end{array}\right],\\ \overset{n}{\underset{i=1}{\sum }}c_{i}P^{i}+1=0,\\ \left[\begin{array}{ccccc} P_{1}^{-1} & P_{2}^{-1} & P_{3}^{-1} & \cdots & P_{s}^{-1}\\ P_{1}^{-2} & P_{2}^{-2} & P_{3}^{-2} & \cdots & P_{s}^{-2}\\ \cdot & \cdot & \cdot & \cdot & \cdot \\ \cdot & \cdot & \cdot & \cdot & \cdot \\ P_{1}^{-s} & P_{2}^{-s} & P_{3}^{-s} & \cdots & P_{s}^{-s} \end{array}\right]\left[\begin{array}{c} R_{1}\\ R_{2}\\ \cdot \\ \cdot \\ R_{s} \end{array}\right]=-\left[\begin{array}{c} m_{0}\\ m_{1}\\ \cdot \\ \cdot \\ m_{q-1} \end{array}\right]. \end{array}$$

## 4. T-WCAWE Using Maximum Likelihood Scheme

In T-WCAWE model, a correction term *J*_*u*_ is introduced to obtain more stable responses. This correction term *J*_*u*_ is calculated from *Z* matrix. The *Z* matrix is computed from ill-conditioned moments and well-conditioned moments, as shown in (16). This *Z* matrix is nonsingular [13, 14]. The correction term *J*_*u*_ is obtained by using maximum likelihood (ML) from

(20) $$\begin{array}{c} J_{u}\left(i\right)=\overset{r}{\underset{i=1}{\prod }}Z_{\left[i:\;i+2q+w-1,\;\;i:\;i+w-1\right]}^{-1}, \end{array}$$

where *r* is the number of nodes, *w* is the order of the equation, and *q* is the order of the product. Now, the new T-WCAWE poles and residues are calculated from (21) and (22), respectively:

(21) $$\begin{array}{c} P^{*}\left(i\right)=J_{u}\left(i\right)P\left(i\right), \end{array}$$

(22) $$\begin{array}{c} R^{*}\left(i\right)=J_{u}\left(i\right)R\left(i\right). \end{array}$$

## 5. TWCAWE with Mass Effect Scheme

In partitioning of FEM, it was reported that the resulting interpolation function can lead to singular or ill-conditioned. This singularity occurs because some interpolations are linearly dependent and ill-condition occurs because the functions are very close to each other. To minimize this singularity and ill-condition, the mass matrix is changed by introducing a factor “*ς*” [18]. For small value of *ς*, TWCAWE-ME scheme reduces the effect of mass, as given in

(23) $$\begin{array}{c} D_{s}=\left(1-\varsigma \right)D_{c}+\varsigma D_{L}, \end{array}$$

where 0 ≤ *ς* ≤ 1.

Here,

(24) $$\begin{array}{c} D_{c}=\frac{\kappa {\left(\bar{\Delta x}\right)}^{2}}{36}\left[\begin{array}{cccc} 4 & 2 & 1 & 2\\ 2 & 4 & 2 & 1\\ 1 & 2 & 4 & 2\\ 2 & 1 & 2 & 4 \end{array}\right],\\ D_{L}=\frac{\kappa {\left(\bar{\Delta x}\right)}^{2}}{4}\left[\begin{array}{cccc} 1 & & & \\ & 1 & & \\ & & 1 & \\ & & & 1 \end{array}\right], \end{array}$$

where *κ* is the grid aspect ratio and Δ*x* is the length of each element in *x* axis. *D*_*c*_ and *D*_*L*_ are consistent and row sum-lumped mass matrices, respectively. The lumped mass matrix *D*_*L*_ is calculated using the total mass and allocating the lumped masses in the ratio of the diagonal element of the consistent mass matrix [19]. In this scheme, *D* from (5) is replaced by new calculated *D*_*s*_.

### 5.1. Transient Response

The final transient response for any arbitrary node *i* is

(25) $$\begin{array}{c} T_{i}\left(t\right)=\text{zsr}\left(t\right)+\text{zir}\left(t\right), \end{array}$$

where

(26) zsr(t)=∑i=1qRi∗Pi∗[{e(real(Pi∗t)   ×(cos⁡(imag(Pi∗))t     +sin(imag(Pi∗))t)}−1],zir(t)=∑i=1qPi∗{e(real(Pi∗t)   ×(cos⁡(imag(Pi∗))t     +sin(imag(Pi∗))t)}.

## 6. Results Analysis

**Figure 1:**
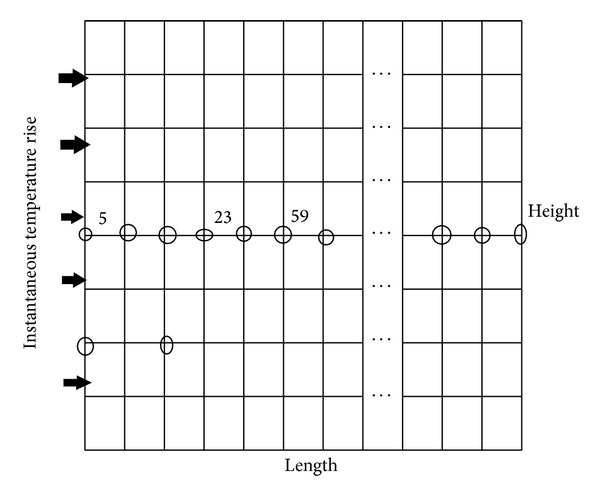
Rectangular two-dimensional slab.

In the present study, the non-Fourier heat conduction is analyzed by considering two-dimensional rectangular configurations using FEM and Tikhonov based WCAWE algorithm. The simulation work was carried out on a rectangular two-dimensional slab meshed to generate the rectangular elements, as shown in Figure 1 where the instantaneous boundary condition was applied at the left boundary of the slab. Boundary condition employed for this simulation work is given as

(27) $$\begin{array}{c} \varphi =\frac{\theta -\theta_{0}}{\theta_{w}-\theta_{0}}, \end{array}$$

where *θ*_*w*_ = rise of temperature at the left boundary edge from the initial value *θ*_*o*_. The applied normalized boundary conditions are stated as follows:

(28) $$\begin{array}{c} \varphi \left(0,\varsigma ,\psi \right)=1,\;\;\frac{\partial \varphi }{\partial \xi }\left(1,\varsigma ,\psi \right)=0,\\ \frac{\partial \varphi }{\partial \psi }\left(\varsigma ,1,\psi \right)=0,\;\;\frac{\partial \varphi }{\partial \psi }\left(\varsigma ,0,\psi \right)=0. \end{array}$$

**Figure 2:**
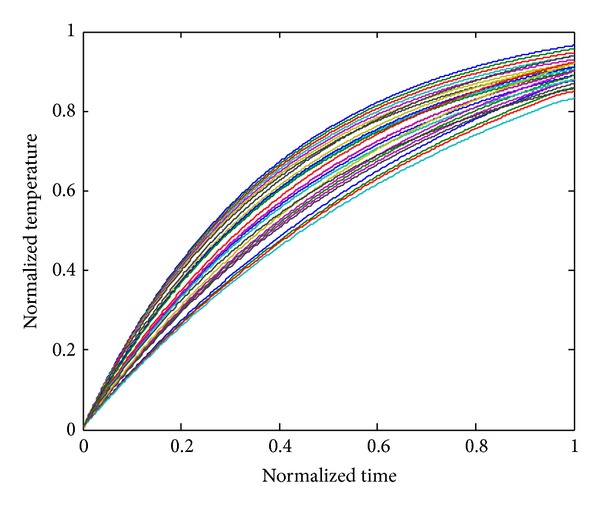
Normalized temperature response spectrum along the center of the slab in the case of *Z*_*T*_ = 0.5.

**Figure 3:**
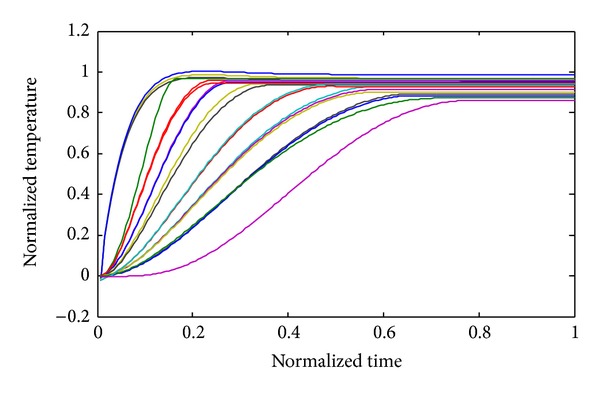
Normalized temperature response spectrum along the center of the slab in the case of *Z*_*T*_ = 0.05.

**Figure 4:**
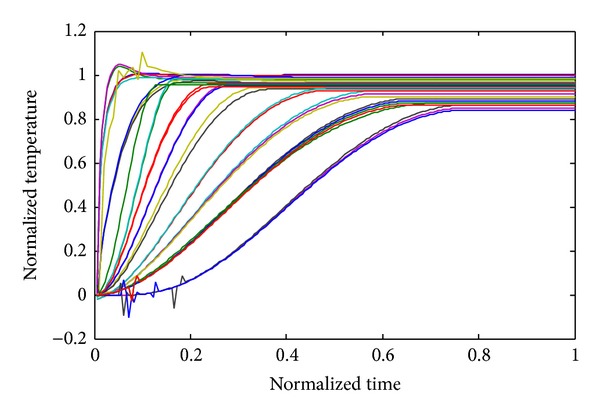
Normalized temperature response spectrum along the center of the slab for the case of *Z*_*T*_ = 0.0001.

Figures 2, 3, and 4 show the temperature distribution for the above rectangular slab considering different values of *Z*_*T*_ as 0.5, 0.05, and 0.0001, respectively. The temperature spectrum shown in Figures 2–4 is plotted by considering the node situated in the middle of the slab; all the responses are calculated by TWCAWE-ML scheme. It is clear from the figure that all temperature responses are continuous and accurate for different values of *Z*_*T*_. In this scheme, the individual poles and residues are used to calculate the individual temperature responses. Hence, this scheme accurately predicts the temperature distribution across the system. The initial high frequency and delay due to relaxation time of electron are clear in Figure 4 for *Z*_*T*_ = 0.0001.

**Figure 5:**
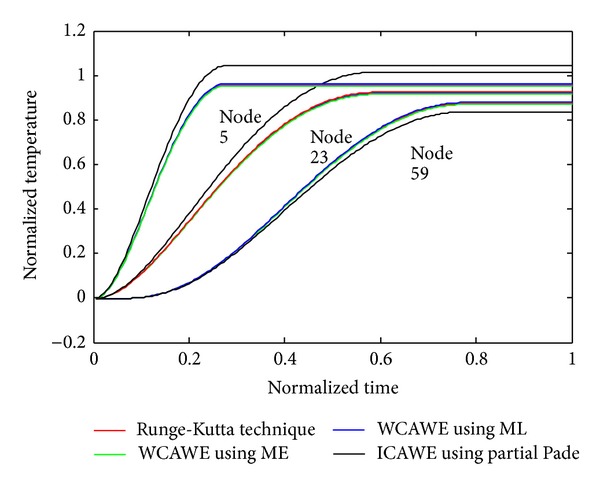
Comparison with TWCAWE-ML, TWCAWE-ME, Runge-Kutta technique, and AWE using partial Pade in the case of *Z*_*T*_ = 0.05.

**Figure 6:**
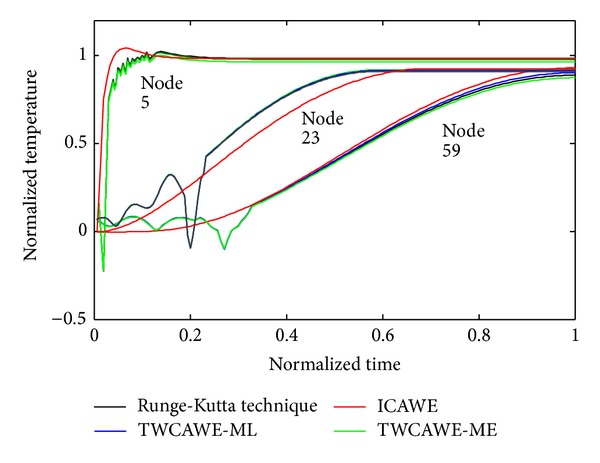
Comparison of TWCAWE-ML, TWCAWE-ME, Runge-Kutta technique, and ICAWE for the case of *Z*_*T*_ = 0.0001.

Though the fourth-order Runge-Kutta technique is slow, the results obtained from this method are more accurate due to its stability. Therefore, our proposed model is compared with the results obtained by Runge-Kutta technique in Figures 5 and 6.

To show the accuracy of our proposed model, we compared both schemes, TWCAWE-ML and TWCAWE-ME, against AWE using partial Pade and Runge-Kutta technique, as shown in Figure 5. The comparison has been presented with *Z*_*T*_ = 0.05. The temperature behavior obtained from both schemes of TWCAWE is similar to Runge-Kutta technique. Partial Pade AWE, however, is unable to predict the temperature behavior as Runge-Kutta technique. In partial Pade AWE, arbitrarily chosen poles and residues are used to approximate the temperature behavior for the whole system. Hence, this method was unable to calculate the temperature behavior for other nodes. On the other hand, both schemes of TWCAWE model use its own poles and residue to calculate the temperature behavior.

The comparison between our proposed two schemes is shown in Figure 6 where *Z*_*T*_ = 0.0001. TWCAWE-ME is able to calculate the delay and high frequency, but the temperature behavior deviates from Runge-Kutta results. On the other hand, the temperature behavior obtained from TWCAWE-ML is in close proximity to Runge-Kutta technique.

**Figure 7:**
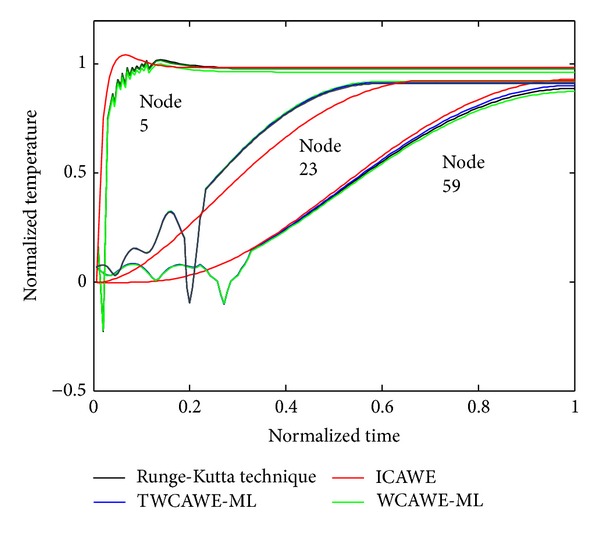
Comparison of TWCAWE-ML, WCAWE-ML, and ICAWE for *Z*_*T*_ = 0.0001.

To bring out the effectiveness of employing Tikhonov technique, we have performed another comparison in Figure 7 in the case of *Z*_*T*_ = 0.0001. This figure shows that the temperature behavior obtained from AWE technique largely deviates from Runge-Kutta behavior. In AWE model, unstable poles make moment matching process ill conditioned.

The result of the WCAWE-ML model is not similar to Runge-Kutta technique due to the singularity problem of the stiffness matrix as shown in Figure 7. The error is compounded during moment calculation and it becomes more evident for higher order moment calculation. This causes WCAWE-ML to be unstable, thus producing inaccurate initial high frequency responses, making it difficult to observe the delay. However, by embedding Tikhonov technique into existing WCAWE-ML, singularity problem was minimized. This is carried out by adding a new term based on Tikhonov regulation technique as given in (13). The new term reduces the symmetrical nature of stiffness matrix, thus minimizing the singularity problem with negligible effect on the temperature behavior. By observing temperature behavior on node 5 which is near the imposed boundary condition, Tikhonov based WCAWE-ML (TWCAWE-ML) is in close proximity to Runge-Kutta technique, as shown in Figure 7, compared to WCAWE-ML which is devoid of Tikhonov technique. The model proposed in the present work is able to reduce the effect of singularity as well as instability of the previous model.

Table 1 shows the computational time required by analyzing different methods in the present work. From the table, TWCAWE-ML is found to be almost two times faster than Runge-Kutta technique. The orthonormalization and maximum likelihood approximation process in T-WCAWE poses computational load that makes it slower compared to AWE or partial Pade AWE. As TWCAWE-ME does not require orthonormalization and maximum likelihood approximation, it performs as fast as AWE or AWE using partial Pade despite some sacrifice in temperature response quality in comparison to TWCAWE-ML. However, TWCAWE-ME showed more stable and hence accurate results compared to either AWE or AWE using partial Pade.

## 7. Conclusion

In the present work, the heat conduction based on non-Fourier model has been solved using FEM. The phase lag responsible for finite relaxation time is varied to analyze the capability of our proposed model for predicting the heat conduction in two-dimensional model. From the results, both schemes discussed in this work are able to reduce the instability of AWE. From the comparison, we can conclude that T-WCAWE using ML results is in close proximity to Runge-Kutta technique compared to TWCAWE-ME. However, TWCAWE-ME scheme is 3.3 times faster than the conventional solver, namely, Runge-Kutta technique (RK4), and needs 1.7 times less computational time than T-WCAWE with ML scheme. Despite this, the TWCAWE-ML is more accurate for predicting the initial frequency as well as delay based on the close proximity to Runge-Kutta technique, hence emerging as the most stable and accurate technique to analyze the fast transient thermal analysis of non-Fourier heat conduction model when compared to AWE, partial Pade AWE, and TWCAWE-ME.

## Figures and Tables

**Table 1:** Required time for different methods.

Method	Time (s)	Ratio with respect to RK4
Runge-Kutta	16	1
T-WCAWE with ML	8.2	1.95
T-WCAWE with ME	4.8	3.33
ICAWE	4.8	3.33
ICAWE with partial Pade	4.8	3.33

## Nomenclature

*R*: Residue
*M*: Moment
*P*: Pole
*φ*: Dimensionless temperature
*τ*: Dimensionless time
*ς*: Distance along “*x*”
*ψ*: Distance along “*y*”
*N*: Shape function matrix
*A*: Element area
*θ*_*o*_: Initial temperature
*θ*_*w*_: Raised temperature
*l*: Length
*h*: Width
*Z*_*T*_: Phase lag for temperature gradient
*Z*_*q*_: Phase lag for heat flux.
